# Severe Autoimmune Hemolytic Anemia Following Pneumococcal Vaccination in an Infant With Underlying Glucose-6-Phosphate Dehydrogenase (G6PD) Deficiency: Report of a Case and Review of the Literature

**DOI:** 10.7759/cureus.68552

**Published:** 2024-09-03

**Authors:** Panagiotis Panagopoulos, Konstantina Kapetaniou, Despoina Gkentzi, Christos Dionysios Biliris, Ekaterini Siomou, Alexandros Makis

**Affiliations:** 1 Department of Pediatrics, University Hospital of Ioannina, Faculty of Medicine, School of Health Sciences, University of Ioannina, Ioannina, GRC; 2 Department of Pediatrics, University Hospital of Patras, Patras, GRC

**Keywords:** urinary tract infection, e coli: escherichia coli, vaccination, glucose-6-phosphate dehydrogenase deficiency, autoimmune hemolytic anemia

## Abstract

Autoimmune hemolytic anemia (AIHA) and glucose-6-phosphate dehydrogenase (G6PD) deficiency are two distinct causes of hemolysis in children and a combination of both diseases is considered rare, especially in early infancy. We present such a rare case of severe AIHA in an infant with G6PD deficiency in the setting of Escherichia coli urinary tract infection and recent pneumococcal vaccination history, with the goal of analyzing potential links between them, examining the causative role of vaccines, and reviewing available literature.

## Introduction

Autoimmune hemolytic anemia (AIHA) refers to a spectrum of disorders that result in the production of autoantibodies against autoantigens on the surface of red blood cells (RBC), eventually leading to their premature destruction (hemolysis) [1].

Based on the antibody types in the direct antiglobulin test (DAT), four types of AIHA have been described in childhood: warm AIHA, which is often IgG+ or IgG+/C3d+; cold agglutinin disease, which is often IgM+/C3d+; paroxysmal cold hemoglobinuria, which is often C3d+ with the presence of Donath-Landsteiner antibody; and mixed type [2]. However, up to 11% of AIHAs are DAT-negative, and this percentage can be minimized in highly specialized labs with the use of more sensitive DAT assays [3].

Based on pathogenesis, there are two types of AIHA: primary or idiopathic, whose causes are unknown; and secondary, usually caused by infections, autoimmune diseases, malignancies, or drugs such as α-methyldopa and penicillin. Infectious factors that can induce AIHA are, among others, Epstein-Barr virus (EBV), cytomegalovirus (CMV), parvovirus B19, rotavirus, varicella-zoster virus, adenovirus, enterovirus, Escherichia coli, and mycoplasma pneumoniae while the most common autoimmune causes of AIHA include systemic lupus erythematosus, Sjögren’s disease, scleroderma, hepatitis and thyroiditis. Hodgkin’s and non-Hodgkin’s lymphoma, B-cell acute lymphoblastic and myeloid leukemia, and chronic myelogenous and lymphoblastic leukemia account for the majority of malignant causes. Primary immunodeficiencies (PIDs), such as common variable immunodeficiency, autoimmune lymphoproliferative syndrome, and adenosine deaminase deficiency have also been reported as causes of secondary AIHA, mainly in young ages and infancy [1-4]. Vaccines, especially vaccines against diphtheria-tetanus-pertussis and severe acute respiratory syndrome-coronavirus-2 (SARS-CoV-2) could be another possible cause of AIHA, which, however, remains unproven. Infections and vaccinations as causative agents of AIHA mainly in childhood [5-11].

Pediatric AIHA is considered to be a disease of young children and has an incidence rate of 3 cases per 1 million children while its mortality ranges widely from 4% to 30% [1,2].

As opposed to pediatric AIHA, G6PD deficiency is a common, especially in the Mediterranean basin, X-linked genetic disorder caused by an inherited defect of the enzyme G6PD in RBCs, which is normally involved in glucose metabolism and whose main function is the production of nicotinamide adenine dinucleotide phosphate (NADPH). NADPH is then used by reductive systems to protect RBCs from oxidative stress. G6PD deficiency has been suggested, though not proven, as both an aggravating and a causal factor for AIHA [12].

## Case presentation

A three-month-old male infant was admitted due to a two-day history of pallor and reduced milk intake. The infant was not breastfeeding. No fever, drug intake, or exposure to extreme circumstances were reported. Vaccination with pneumococcal 13-valent conjugate vaccine was performed 4 days before admission. The hexavalent vaccine had been administered at the age of two months. Apart from the G6PD deficiency found in newborn screening (G6PD was measured at 4.3 U/g Hb), the past medical and perinatal history was unremarkable, with normal growth and psychomotor development. Regarding the family history, the parents were not related and both the mother and the older two-year-old brother have G6PD deficiency.

**Figure 1:**
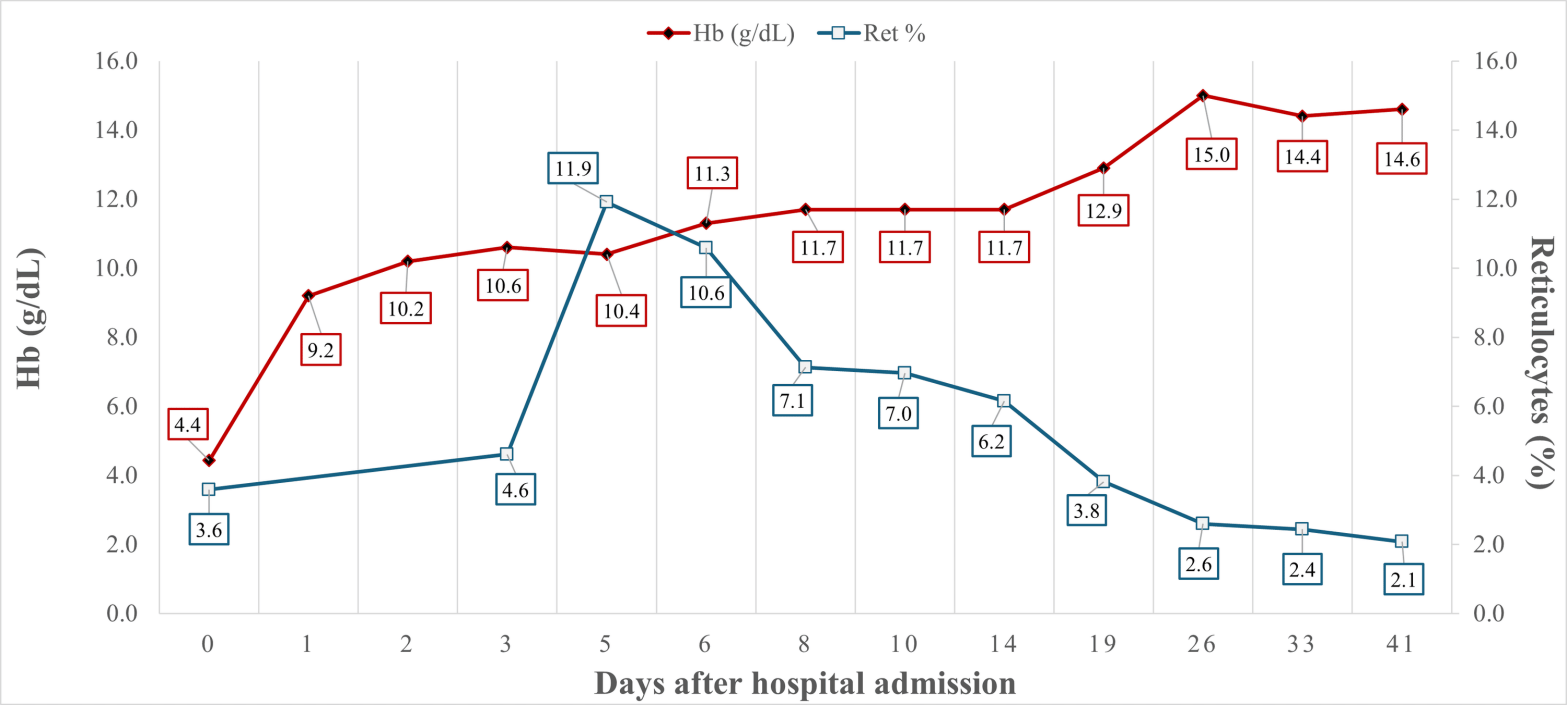
Hemoglobin (g/dl) and reticulocytes (%) during the course of the disease

The physical examination revealed a heart rate of 140 beats per minute, tachypnea with a respiration rate of 70 per minute, pallor, and jaundice without organomegaly or lymphadenopathy. Chest X-ray and abdominal ultrasound had normal findings. As shown in Table 1, severe anemia was confirmed by the laboratory tests, with hemoglobin 4.43 g/dL and 3.59% reticulocytes (corrected 1.4%). The possible explanations for the extremely low reticulocyte count are the immune effect of autoantibodies against the erythrocyte marrow precursors, the temporary suppression of the hematopoietic activity secondary to infection, or the delayed bone marrow response as shown in Figure 1. The total bilirubin (TBL) was 4.8 mg/dL, mainly unconjugated (4.6 mg/dL) with a high LDH of 1072 U/L, thus indicating hemolytic anemia. Liver transaminases were raised, with an aspartate aminotransferase (AST) of 159 IU/L and an alanine aminotransferase (ALT) of 95 IU/L. An elevation in white blood cells (23.10 x 103/μL, 50% lymphocytes) and platelet count (635 x 103/μL) was also noted. Blood film showed macrocytosis with spherocytes, polychromasia, 2% nucleated RBCs, rouleaux formation, immature granulocytes, and toxic granulation of neutrophils as well as on cells with blastic characteristics. DAT was positive for IgG (3+), IgA (2+), IgM (3+) and C3d (3+). The cold antibody titer was low, and the Donath-Landsteiner test was negative, confirming the diagnosis of warm-type AIHA. A urine culture obtained through suprapubic aspiration revealed Escherichia coli > 105 cfu/mL, susceptible to amoxicillin/clavulanate, which was started on the patient. We performed suprapubic aspiration due to presumptive evidence of a urinary tract infection from the infant’s urinalysis: special gravity 1006, pH 6, nitrate negative, 7-10 WBC/hpf, 0-3 RBC/hpf, several microorganisms.

**Table 1:** Laboratory findings during hospitalization and follow-up WBC: white blood cells, Hb: hemoglobin, PLT: platelets, RBC: red blood cells, MCV: mean corpuscular volume, MCH: mean corpuscular hemoglobin, Ret: reticulocytes, DAT: direct antiglobulin test, ALT: alanine transaminase, LDH: lactate dehydrogenase, CRP: C-reactive protein

Parameter	Reference Range	Admission	3 days	6 days	3 weeks	6 weeks	6 months
WBC	4-10×10^9^/L	23.10	8.81	13.97	9.88	15.02	10.11
Hb	8.2-13.8 g/dL	4.43	10.4	11.3	12.9	14.6	13.2
PLT	150-400×10^9^/L	635	429	297	581	420	343
RBC	4.00-6.60×10^12^/L	3.43	3.56	3.69	4.2	4.67	4.71
MCV	80-100 fl	85	95.8	90.2	93.9	91.9	87.5
MCH	27-31 pg	28.44	31.5	30.6	31.5	31.3	28
Ret	0-2 %	3.59 %	11.91 %	10.59 %	3.81 %	2.08 %	1.69%
DAT		Positive	Positive	Positive	Ambiguous	Negative	Negative
ALT	10-35 IU/L	95	70	69	57	71	16
Total bilirubin	<1.2 mg/dL	4.8	0.53	0.5	0.4	0.5	0.4
Direct bilirubin	<0.2 mg/dL	0.2	0.1	0.08	0.07	0.1	0.07
Urea	10-40 mg/dL	57	20	22	20	24	15
LDH	115-230 IU/L	1072	544	471	319	446	298
CRP	mg/dL	7	2	3	2	2	1

Due to severe AIHA, the infant received three RBC transfusions (5 mL/kg - 6 mL/kg - 7 mL/kg, respectively), and intravenous methylprednisolone was started with a dose of 1 mg/kg every 6 hours. A bone marrow aspiration was performed due to the finding of immature white blood cells in the peripheral smear as well as the very low reticulocyte count. The bone marrow sample was reported as suboptimal because there were not enough marrow particles or megakaryocytes. No blastic cells were found. The bone marrow immunophenotyping and cytogenetic analysis were normal. Frozen serum was stored to investigate the cause of the AIHA. A rapid rhinopharyngeal antigen test for SARS-CoV-2 was negative. On the third day, the patient was stable, the hemoglobin reached 10.4 g/dl, and treatment was continued with oral prednisolone 2 mg/kg/day divided into 3 doses. This dose was administered for four weeks with gradual normalization of hemoglobin, reticulocytes, lactate dehydrogenase (LDH), TBL, and negative DAT as shown in Table 1. During the following two months, prednisolone was slowly and gradually tapered to one daily dose until the dose was below 1 mg/kg/day, with normal findings regarding the hemoglobin and the hemolysis parameters (Figure 1). For the next 2 months, prednisolone was gradually tapered to 0.5 mg/kg/day. At this point, at the age of five months, it was decided to administer the second dose of the hexavalent vaccine against diphtheria, tetanus, pertussis, poliomyelitis, hepatitis B virus, and Hemophilus influenzae type b. One month later, at the age of 6 months, while the infant was on daily prednisolone, the second dose of the pneumococcal 13-valent conjugate vaccine was administered. Both vaccinations were uneventful, without any negative effects on the hematological profile. After that, prednisolone was given every other day for two weeks and was finally stopped with normal laboratory tests, and the DAT was negative.

The stored serum was used to measure IgM levels against CMV, EBV, and parvovirus B19, which were negative. Due to the presentation of AIHA in infancy, we had to exclude systemic lupus erythematosus (SLE) and PIDs. Antinuclear and anti-(double-stranded)-DNA antibodies were negative. Immunoglobin (IgG, IgM, IgA) levels were within the age-appropriate range. Furthermore, a whole exome sequencing (WES) analysis was negative for PIDs but revealed a heterozygous state for Gaucher disease (c.1226A>G, N370S), which was irrelevant to the clinical picture of the patient. WES also showed the G6PD Mediterranean variant (c.653C>T (p.Ser218Phe)) and confirmed the diagnosis of G6PD deficiency.

## Discussion

Pediatric AIHA has a wide range of known triggers such as infections, autoimmune diseases, PIDs, and malignancies. The differential diagnosis becomes even harder when unproven triggers, such as vaccination and G6PD deficiency, are taken into consideration. Regarding our patient, we were able to exclude PIDs, autoimmune diseases, malignancies, and the most common viral triggers of AIHA, which led to the conclusion that AIHA was caused by one or more out of three; the Escherichia coli urinary tract infection, the G6PD deficiency, or the vaccination with pneumococcal 13-valent conjugate vaccine.

As far as Escherichia coli infection is concerned, it is considered a causative factor for AIHA and has been described as such in studies. A nationwide French observational study described three cases of Escherichia coli-induced AIHA, accounting for 6% of the infectious causes [1] while a single-center retrospective study reported one case of warm AIHA in an infant, which was caused by Escherichia coli infection [4]. Therefore, it was placed high on the list of our differential diagnoses.

The role of G6PD deficiency in AIHA is, on the other hand, unclear. However, there have been four case reports of simultaneous presentation of AIHA and G6PD deficiency [13-16]. The ages of the patients ranged between 10 and 21 years. One of the cases was associated with reported aspirin intake and another one with acute hepatitis A [13,15]. The mechanism by which G6PD deficiency affects hemolysis remains unknown. A study in mice suggested that oxidative stress causes increased red blood cell phagocytosis, which results in easier recognition of oxidized surface proteins as autoantigens, provoking autoimmunity [13]. As G6PD deficiency is known to aggravate the normal oxidative stress that red blood cells undergo, it could function as a co-factor in the development and severity of AIHA.

Finally, vaccination has also been associated with AIHA, and mechanisms for this have been suggested. Eight cases of AIHA following DTP vaccination with a simultaneous absence of other known causative factors have been reported [5,7-11]. Our search revealed a single case report suggesting a possible link between pneumococcal vaccination in a 72-year-old man and cold AIHA [17]. Moreover, numerous cases of correlation between the SARS-CoV-2 vaccines and AIHA have been described, even though several patients were ultimately diagnosed with B-lymphoid malignancies [6]. Two possible mechanisms of vaccine-induced AIHA have been suggested. On the one hand, vaccines could function as haptens with affinity to the red blood cell membrane [5]. On the other hand, molecular mimicry between vaccine proteins, such as the SARS-CoV-2 spike protein, and proteins on the surface of red blood cells, such as ankyrin-1, has been hypothesized [6].

A possible link of AIHA to vaccination was particularly concerning, as it could be a severe obstacle, regarding the infant’s further vaccination protocol. However, given the protective value of vaccination, it was decided that an AIHA relapse would be less severe than a vaccine-preventable disease. Therefore, both the second dose of the hexavalent vaccine against diphtheria, tetanus, pertussis, poliomyelitis, hepatitis B virus, and Hemophilus influenzae type b as well as the second dose of the pneumococcal 13-valent conjugate vaccine were successfully administered at the age of 5 and 6 months, respectively, while the patient was under low dosages of corticosteroids. The uncomplicated repeated vaccinations probably support a co-existent causative role of the E.coli UTI to the appearance of AIHA in our patient.

## Conclusions

The presentation of our case aimed to emphasize the unique characteristics of AIHA in early infancy. In this age period, the immune system is immature, and the finding of the underlying triggering cause is quite challenging. A large proportion of AIHA in infants is idiopathic. PIDs, as well as infections, autoimmune diseases, and malignancies, must necessarily be excluded. The obligatory and essential vaccinations in infancy further complicate the diagnostic process. The therapeutic approach of AIHA in infants has to be rapid, effective, and with the least toxicity possible since AIHA can be particularly severe, and refractoriness is common at this age. The presence of other medical conditions that aggravate the oxidative stress that already exists in AIHA, can make the overall management quite demanding, as was G6PD deficiency in our case, which is common in Mediterranean countries. In our case, apart from the G6PD deficiency, two other factors were taken into account: the Escherichia coli urinary tract infection and vaccination with pneumococcal 13-valent conjugate vaccine. To conclude, although the co-existence of AIHA and G6PD deficiency rarely occurs in children, it can potentially worsen the severity of the autoimmune process, especially if combined with infections and other medical conditions such as vaccinations. Particularly when vaccination is suggested as a possible cause, it is important that clinicians plan a therapeutic approach to minimize complications and prevent future vaccine avoidance due to parental fear.
